# A Non-Instrumental Green Analytical Method Based on Surfactant-Assisted Dispersive Liquid–Liquid Microextraction–Thin-Layer Chromatography–Smartphone-Based Digital Image Colorimetry(SA-DLLME-TLC-SDIC) for Determining Favipiravir in Biological Samples

**DOI:** 10.3390/molecules28020529

**Published:** 2023-01-05

**Authors:** Bharti Jain, Rajeev Jain, Prashant Kumar Jaiswal, Torki Zughaibi, Tanvi Sharma, Abuzar Kabir, Ritu Singh, Shweta Sharma

**Affiliations:** 1 Central Forensic Science Laboratory, Dakshin Marg, Sector—36A, Chandigarh 160036, India; 2 Institute of Forensic Science & Criminology, Panjab University, Chandigarh 160014, India; 3 School of Earth Sciences, Department of Environmental Sciences, Central University of Rajasthan, NH-8, Bandar Sindri, Ajmer 305817, India; 4 Department of Medical Laboratory Sciences, Faculty of Applied Medical Sciences, King Abdulaziz University, Jeddah 21589, Saudi Arabia; 5 King Fahd Medical Research Center, King Abdulaziz University, Jeddah 21589, Saudi Arabia; 6 International Forensic Research Institute, Department of Chemistry and Biochemistry, Florida International University, Miami, FL 33199, USA; 7 Department of Pharmacy, Faculty of Allied Health Science, Daffodil International University, Dhaka 1207, Bangladesh

**Keywords:** favipiravir, surfactant-assisted dispersive liquid–liquid microextraction, digital image colourimetry, thin-layer chromatography

## Abstract

Favipiravir (FAV) has become a promising antiviral agent for the treatment of COVID-19. Herein, a green, fast, high-sample-throughput, non-instrumental, and affordable analytical method is proposed based on surfactant-assisted dispersive liquid–liquid microextraction (SA-DLLME) combined with thin-layer chromatography–digital image colourimetry (TLC-DIC) for determining favipiravir in biological and pharmaceutical samples. Triton X-100 and dichloromethane (DCM) were used as the disperser and extraction solvents, respectively. The extract obtained after DLLME procedure was spotted on a TLC plate and allowed to develop with a mobile phase of chloroform:methanol (8:2, *v*/*v*). The developed plate was photographed using a smartphone under UV irradiation at 254 nm. The quantification of FAV was performed by analysing the digital images’ spots with open-source ImageJ software. Multivariate optimisation using Plackett–Burman design (PBD) and central composite design (CCD) was performed for the screening and optimisation of significant factors. Under the optimised conditions, the method was found to be linear, ranging from 5 to 100 µg/spot, with a correlation coefficient (R^2^) ranging from 0.991 to 0.994. The limit of detection (LOD) and limit of quantification (LOQ) were in the ranges of 1.2–1.5 µg/spot and 3.96–4.29 µg/spot, respectively. The developed approach was successfully applied for the determination of FAV in biological (i.e., human urine and plasma) and pharmaceutical samples. The results obtained using the proposed methodology were compared to those obtained using HPLC-UV analysis and found to be in close agreement with one another. Additionally, the green character of the developed method with previously reported protocols was evaluated using the ComplexGAPI, AGREE, and Eco-Scale greenness assessment tools. The proposed method is green in nature and does not require any sophisticated high-end analytical instruments, and it can therefore be routinely applied for the analysis of FAV in various resource-limited laboratories during the COVID-19 pandemic.

## 1. Introduction

The outbreak of the coronavirus represents one of the most dreadful viral diseases which has been endangering the lives of millions of people [1]. As a result, the World Health Organization declared the outbreak a pandemic in March 2020. However, the number of infections is still increasing to unprecedented levels [2]. Therefore, the immediate urge to identify new therapeutics to combat the COVID-19 pandemic prompted the development of several possible drugs, including hydroxychloroquine, ivermectin, remdesivir, and favipiravir (FAV) [3,4]. The results of the clinical trials revealed that favipiravir was a potential COVID-19 treatment option, as it ameliorated signs and symptoms and enhanced viral clearance [5].

**Figure 1:**
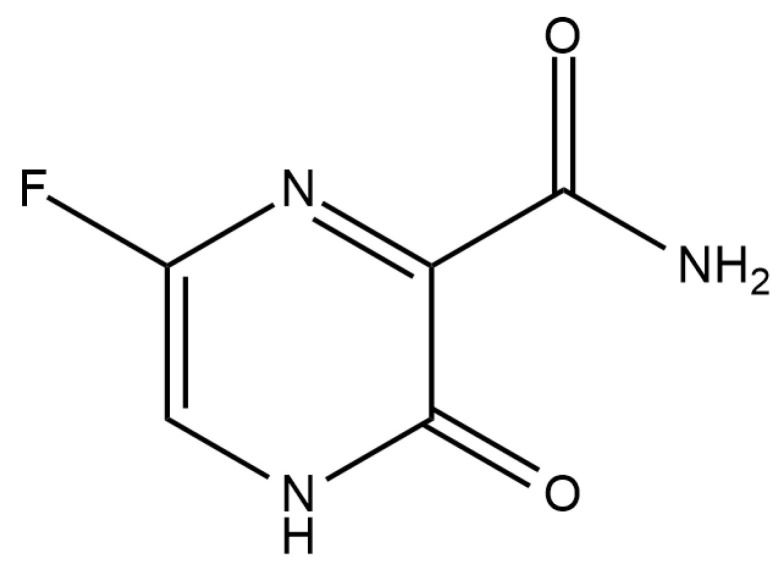
Chemical structure of favipiravir.

As depicted in Figure 1, the purine nucleoside precursor FAV (6-fluoro-3-oxo-3,4- dihydropyrazine -2-carboxamide) is a pyrazine carboxamide derivative providing potent antiviral activity against a variety of RNA viruses [6]. In 2014, Fujifilm Toyama Chemical Company was the first to develop FAV as a treatment for influenza in Japan. After being consumed, the medication is absorbed into the body and assimilates into the cells, where it is ribosylated and phosphorylated by the host’s cellular enzymes to produce the active metabolite T-705-ribofuranosyl-5’-triphosphate (T-705-RTP) [7,8]. Then, T-705-RTP integrates into the viral RNA in small amounts and preferentially inhibits the transcription and replication of the RNA-dependent RNA polymerase (RdRp) enzyme of influenza and many other RNA viruses [9,10].

Despite their importance in clinical controls, very few analytical techniques for the quantitative analysis of FAV have been reported. Electrochemical sensors [11,12,13], spectrofluorimetric methods [1,14], reverse-phase high-performance thin-layer chromatography (RP-HPLC) [15], liquid chromatography with tandem mass spectrometry (LC-MS/MS) [4,16,17,18,19], and ultrahigh-performance liquid chromatography with tandem mass spectrometry [20] have been used to detect FAV in pharmaceutical products and biological matrices. Although these methods offer sufficient sensitivity, their high cost of analysis, need for bulky and sophisticated instruments, time consumption, and unsuitability for onsite detection are some of the major constraints preventing them from being of routine use in resource-limited settings during the COVID-19 pandemic.

In order to identify trace levels of these drugs in complex matrices, sample preparation plays a crucial role prior to instrumental analysis [21]. Today, sample preparation techniques tend to emphasise principles of green analytical chemistry (GAC). The main objective of GAC is the advancement of new-generation analytical methods with the purpose of reducing reagent consumption (possibly using biodegradable and low-toxicity solvents), minimising waste generation, consuming the least amount of energy, and ensuring operator/analyst safety, along with the automation and miniaturisation of the analytical process [22]. Dispersive liquid–liquid microextraction (DLLME) has garnered a lot of interest from analysts due to its ease of use, affordability, environmental friendliness, high enrichment factors, and quick extraction capabilities [23,24,25]. DLLME is performed after immediate injection of the extraction and disperser solvents into an aqueous phase. As a result, a cloudy solution is formed, consisting of small extraction solvent droplets scattered throughout the aqueous phase. The dense extraction solvent obtained by centrifugation settles as the sedimented phase and is used for further analysis [26]. DLLME has grown in popularity, as indicated by the growing number of applications in fields such as forensic, clinical, environmental, and pharmaceutical analysis, among many others [27,28,29,30,31,32].

TLC is one of the earliest planar chromatographic methods and is still used to separate and identify organic analytes in a mixture [33]. It is regarded as a sustainable chromatographic method owing to its benefits, such as (i) minimal solvent usage; (ii) easy execution; (iii) high sample throughput (i.e., simultaneous analysis of 8–10 samples using the same development solvent); (iv) cost-effectiveness; (v) TLC is based on capillary flow of the solvent and, therefore, requires no pressure controls, pumps, valves, etc., hence entailing no wear and tear and requirement for spare parts; and (vi) no need for specifically trained personnel. Herein, sustainability refers to the probability of system failure and the availability of resources to restore the system to an operational condition. Chromatographic methods are more sustainable when the probability of failure is lower and the availability of restoration resources is higher [34,35]. On the other hand, classical TLC has the limitation of being only a qualitative method. Therefore, quantitative analysis in TLC is carried out by its hyphenation with other detection techniques, such as UV–Vis spectrophotometry, densitometry, FID, and mass spectrometry (MS). However, although these hyphenated techniques are sensitive, they are very expensive and hard to afford in resource-limited settings [36,37,38,39]. 

As an alternative to this, combining TLC with smartphone-based digital image colourimetry (SDIC) can provide a simple, promising, reliable, feasible, and cost-effective alternative for quantitative analysis. Currently, DIC (digital image colourimetry) has attracted considerable interest from researchers to analyse different analytes in pharmaceuticals and to convert images into numerical data. DIC is a kind of colorimetric analysis in which digital images captured by mobile phones, webcams, digital cameras, and scanners are transformed to the RGB colour system, which is composed of three different colour intensities (red, blue, and green). Moreover, it has become a notable research area in analytical chemistry due to its affordability, ease of use, portability, and capacity to analyse data immediately. In comparison to conventional methods, TLC coupled with SDIC offers a number of benefits, such as having the least negative impacts on environment and human health, being able to provide a portable analytical system for a user-friendly experience, offering high sample throughput, energy-efficiency, and no requirement of specifically trained personnel [40,41]. 

Considering the significant burden on analytical laboratories during the COVID-19 pandemic, the present study proposes a novel and green analytical method based on the coupling of SA-DLLME with TLC-SDIC for instrument less detection of FAV in pharmaceutical formulations, as well as human urine and plasma samples. The proposed method does not require any complex apparatus and uses a simple TLC setup, a smartphone camera, and freely available image analysis software. The results obtained by the proposed study were compared with those obtained by the HPLC method for FAV analysis. Furthermore, the green character of the developed method was evaluated using the ComplexGAPI, AGREE, and Eco-Scale greenness assessment tools.

## 2. Results and Discussion

### 2.1. Screening of TLC Parameters

Commonly used solvent systems in routine systematic toxicological analysis—viz., chloroform–acetone (8:2 *v*/*v*), ethyl acetate–ethanol (8:2 *v*/*v*), chloroform–methanol (8:2 *v*/*v*), and ethyl acetate–acetone (8:2 *v*/*v*)—were screened. In order to choose the best solvent system for FAV among the four solvent systems, a series of tests were conducted [42]. The combination of chloroform–methanol (8:2 *v*/*v*) had the best separation for FAV. Additionally, saturation times ranging from 10 to 30 min were also investigated, since this had a substantial impact on the chromatographic separation. A saturation time of 15 min yielded a promising performance. As a result, the chloroform–methanol (8:2 *v*/*v*) combination was chosen as the developing system, with a 15 min saturation period [43]. The R_f_ value was found to be 0.28.

### 2.2. Screening of Surfactant and Extraction Solvent

**Figure 2:**
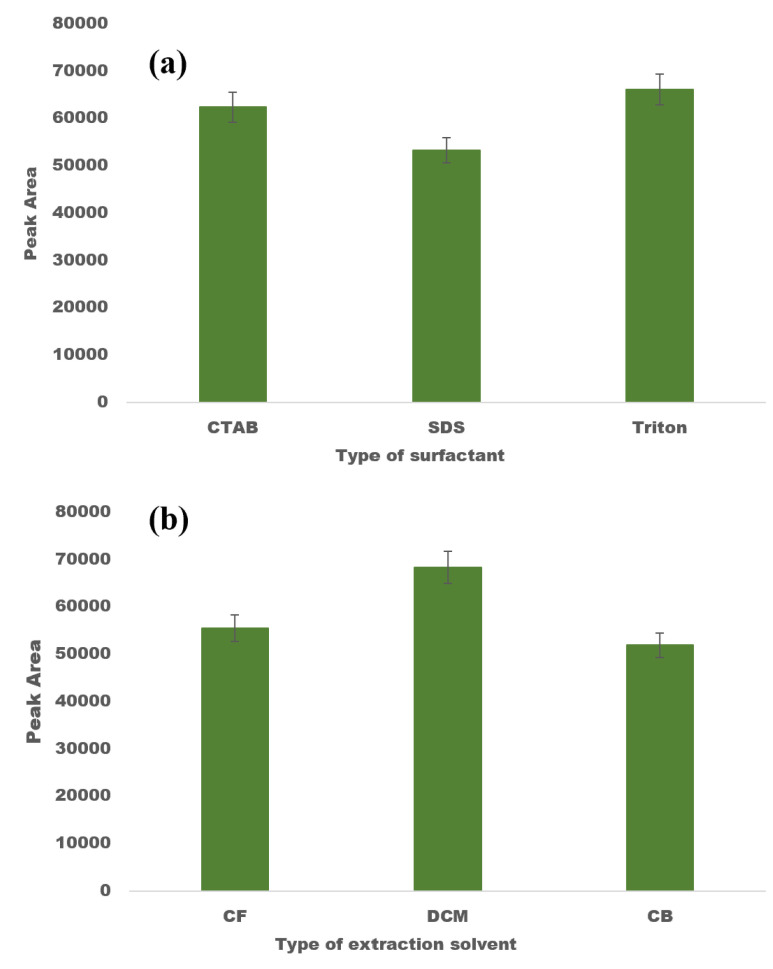
Optimisation of DLLME factors: (**a**) screening of dispersion solvent, and (**b**) screening of extraction solvent.

Prior to performing PBD, the initial experiments were carried out to determine the most suitable surfactant for DLLME. The selected surfactant needs to possess characteristics such as miscibility with both the organic solvent and the aqueous sample, as well as the ability to speed up the emulsification of the organic solvent into the aqueous phase. Owing to their amphipathic structure, surfactants reduce the interfacial tension between two liquids and regulate the hydrophilicity and lipophilicity of the solution [44]. Triton X-100, CTAB, and SDS—three commonly used surfactants—were employed in a number of experiments to find the optimal surfactant to use as the disperser solvent for DLLME. Three different mixtures of 0.045 mmol L^−1^ of disperser solvent (Triton X-100, CTAB, and SDS) along with a constant volume of CF (200 µL) were prepared. With the help of a syringe, this mixture was quickly and forcefully added to an aqueous solution fortified with FAV at 10 µg mL^−1^. At this step, a turbid solution was formed, which was sonicated for 2 min before being centrifuged for 3 min at 5000 rpm. Among all of the tested surfactants, the best extraction efficiency was demonstrated by non-ionic surfactants, i.e., Triton X-100 (Figure 2a). In comparison to ionic surfactants, non-ionic surfactants appeared to have a higher solubilisation capacity and sufficient hydrophobicity for the target analytes. Therefore, Triton X-100 was chosen as the disperser solvent for all further experiments.

The type of extraction solvent directly affects the preconcentration factor and the extraction yield; therefore, its choice is crucial for DLLME. The extraction solvent should be capable of extracting the desired analytes, immiscible in water, and should have a greater density than water. Additionally, it should show good chromatographic properties when spotted on a TLC plate. In accordance to these parameters, the three commonly utilised extraction solvents—viz., DCM, CB, and CF—were evaluated for maximum extraction efficiency for DLLME. For this purpose, a series of experiments were carried out to determine the appropriate extraction solvent among the four solvents tested. Then, 0.045 mmol L^−1^ of Triton X-100 was rapidly injected into the sample solution together with a constant volume of 200 µL of each extraction solvent. Similar to the earlier experiments, all other experimental conditions were identical, and the findings are depicted in Figure 2b. It is evident that employing DCM as an extraction solvent yielded higher recoveries. Therefore, DCM was selected as the suitable extraction solvent.

### 2.3. Multivariate Optimisation

#### 2.3.1. Plackett–Burman Design (PBD)

The selected screening design (i.e., PBD) is a mathematically based statistical tool that can minimise the number of experiments and identify the variables that have an impact on the studied process in order to perform further optimisation [45]. In fact, the current study aimed to enable estimation of the strength of influence of each factor by using Fisher’s test as well as a *p*-value comparison with α risk (α = 0.05). Moreover, the Pareto chart was used to arrange the interactions and effects in decreasing order. This is a quick and effective tool for finding the significant parameters among a large number of factors while minimising the time required and maintaining persuasive data on each variable. In addition, the major effect of each variable was determined as the difference between the average of measurements recorded at the high level (+) and the average of measurements recorded at the low level (−) of that factor. With the help of this, the impact of each factor could be assessed.

For this study, the seven independent factors involved in the PBD were as follows: (i) pH, (ii) ultrasonication time (s), (iii) ionic strength (%), (iv) volume of extraction solvent (µL), (v) volume of disperser solvent (mmol L^−1^), (vi) vortex time (min), and (vii) vortex speed (rpm). For each independent factor, there were two classification levels: (−1) denotes a low level, while (+1) denotes a high level, as shown in Table S1. A 2^7−4^ PBD was employed to identify significant factors. For 24 runs (7 + 1 = 8 × 3 = 24), each experiment was performed in triplicate and in a random order. The peak area was used as a response during the statistical analysis of these factors. In order to analyse the significant parameters, an analysis of variance (ANOVA) test was applied. Additionally, a *t*-test was used to identify significant variables that are represented in the Pareto chart in Figure S1 with a confidence level higher than 95% (*p* < 0.05). As a result, the ultrasonication time, pH, and volume of surfactant act as the disperser solvent were determined to be the most significant variables. In contrast, the vortex speed, vortex time, and volume of the extraction solvent were less significant factors for the extraction of FAV, while the ionic strength was found to be a non-significant factor. In order to further optimise these three most significant variables (ultrasonication time, pH, and volume of surfactant), a central composite design (CCD) of experiments was used.

#### 2.3.2. Central Composite Design (CCD)

A quadratic model was constructed between the dependent and independent variables, using the significant parameters that were identified during the screening procedure. The response surface was used for the study type, whereas the central composite was used for the design type. For the purpose of fitting quadratic polynomials, a CCD incorporates a 2^f^ factorial design with at least one point in the centre of the experimental area to produce rotatability or orthogonality characteristics and additional points such as star points [46]. In this design, the experimental runs were carried out randomly to reduce the impact of uncontrolled variables. For each set of experiments, three independent parameters (pH, ultrasonication time, and volume of surfactant) were specified at three levels (low, centre, and high), with coded values (−1, 0, +1) and star points -α and +α, respectively, as shown in Tables S2 and S3. The total number of experiments (N) was determined to be 18 for these parameters (f = 3) using the following equation:

N = 2^f^ + 2f + N_0_ (1)

The total number of experiments (N) was calculated from eight factorial points (2^f^), six axial points (2f), and four centre points (N_0_). For the CCD, the peak area served as the response. The “goodness of fit” of the acquired results was then evaluated using an ANOVA.

**Figure 3:**
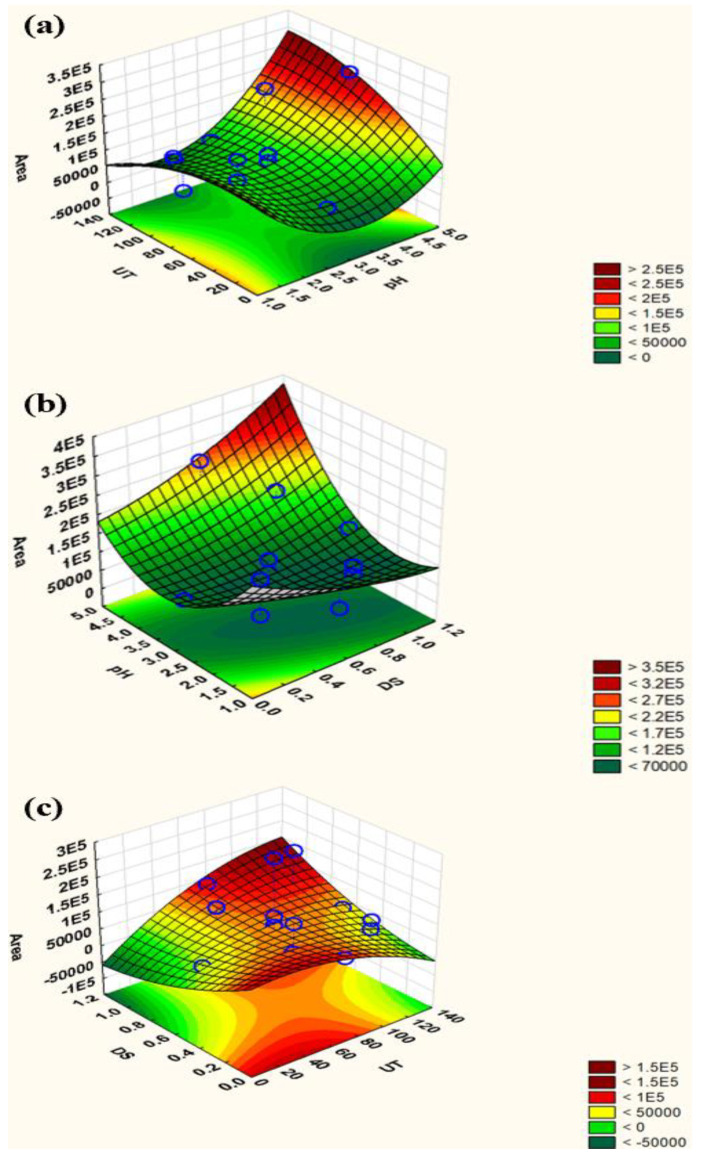
Response surface plots: (**a**) pH vs. ultrasonication time; (**b**) volume of disperser solvent vs. pH; (**c**) ultrasonication time vs. volume of disperser solvent.

The response surface plots of peak area vs. significant factors are depicted in Figure 3, along with the most pertinent fitted response surfaces for the design. The curvatures of these plots represent the interactions of the factors. In addition, desirability function (DF) is a well-known and established tool for simultaneously determining input variables that can provide optimal values for one or more responses. DF provides an easy and quick transformation of various responses into quantitative and qualitative results for a single measurement. The response is converted into a specific desirability function with a range of 0 to 1. Desirability 0 denotes undesirable or minimal circumstances, whereas desirability 1 denotes the maximum. A series of graphs are formed for each independent variable, and a red line shows the resultant optimal value (Figure S2). In this study, the optimal values for these parameters were as follows: 1.13 mmol/L (volume of disperser solvent), 128.67 s (sonication time), and 4.9 (pH). For ease of operation, sonication time and pH were rounded to 130 s and 5, respectively.

### 2.4. Analytical Performance of the Method

Under the optimal conditions, the linearity, accuracy, relative recovery, LODs, and LOQs of the suggested SA-DLLME-TLC-SDIC approach were evaluated. The target analyte (i.e., FAV) was fortified into ultrapure water and biological matrices at different concentrations in the range of 5–100 µg/spot. The proposed method yielded a strong correlation between the concentration and the peak area of the analyte (R^2^ = 0.991–0.994). The ranges of the LODs and LOQs were determined to be 1.2–1.5 µg/spot and 3.96–4.29 µg/spot at signal-to-noise ratios of 3 and 10, respectively. Additionally, the repeatability and reproducibility of the proposed method were assessed using intraday and interday precisions (*n* = 5), which were represented as %RSD. Three distinct concentration levels were used to measure the intraday and interday precisions (%RSD), which were found to be less than 5 and 10%, respectively, as highlighted in Table 1. Furthermore, the enrichment factor (EF), enrichment recovery (ER%), accuracy, and relative recovery (RR%) were also evaluated and are presented in Table 2. The matrix effect (ME, expressed as RR%) was evaluated by utilising five distinct drug-free human plasma and urine samples. This was achieved by comparing the peak area of FAV from post-extracted plasma and urine samples at low, middle and high QC levels to those prepared in pure standards at similar concentrations. The RR% in both matrices was found to be in the range of 87–98% (Table 2), indicating that there was no significant matrix effect on the extraction efficiency of the DLLME procedure.

The preconcentration factor (PF) of the proposed method was found to be 50, as the initial volume of the sample was 10 mL and the final volume of the extract was 0.2 mL. The EF was determined as the ratio of the slope of the calibration curve of the FAV obtained by the proposed method and the slope of the calibration curve of its standard solution. The EF for FAV ranged between 35.1 and 53.9 under optimal conditions. This correlated to ER% findings ranging from 70.2 to 107.8%. Furthermore, in order to evaluate the stability of FAV, low and high-QC samples were used in five replicates. The QC samples were assessed after six freeze–thaw cycles, held at ~4 °C for 12 h, and then thawed separately at room temperature. The variation of accuracy at each level was well within ±15%.

Minor but deliberate changes in the chromatographic process parameters were applied to assess robustness, which was represented as the percentage relative standard deviation (% RSD). Small changes were implemented by altering the composition, the volume of the mobile phase, and the saturation time within a range of ±10%. The outcomes demonstrated that there were no significant changes in the R_f_ values of the FAV, and the %RSD were found to be 0.89%. This indicates that the proposed method is robust and reliable.

### 2.5. Assessment of the Green Character of the Developed Procedure

From the perspective of GAC, evaluating the environmental friendliness of analytical techniques is essential. Since many different parameters are associated with analytical methodologies, it was essential to establish precise metric systems to measure each variable that might pose a risk in terms of its ecological impact on the environment and human safety [47,48]. There are a number of tools that can be helpful for the evaluation of greenness; however, the most well-known ones are the Green Analytical Procedure Index (GAPI), Analytical Eco-Scale, and Analytical GREEnness (AGREE) metrics. Herein, the green character of the proposed analytical method was evaluated using these three prevailing metrics. With the aid of these metrics, the assessment findings are presented in a very readable format.

The Analytical Eco-Scale is the first green assessment tool. This metric evaluates analytical procedures by eliminating penalty points from each stage of the process that does not conform to GAC guidelines. The following equation (Analytical Eco-Scale score = 100—total penalty) is used to calculate penalty points for each of the parameters of the defined procedure, including (i) amounts of reagents, (ii) occupational risks, (iii) waste, and (iv) energy. Table 3 displays the results of calculating the Eco-Scale score for the proposed method. The analytical approach is considered to have excellent greenness if the score is higher than 75. With an Eco-Scale score of 84 (Table 3), the developed approach can be regarded as having outstanding greenness.

**Figure 4:**
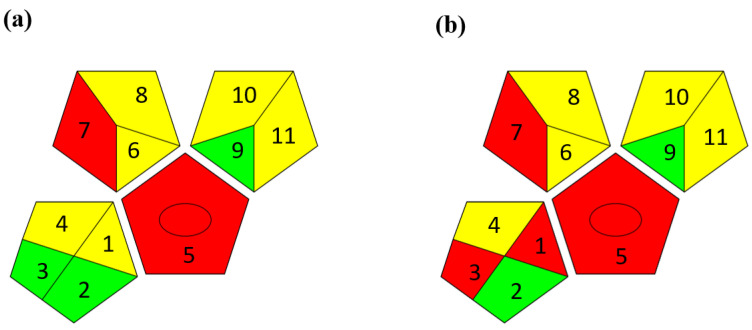
ComplexGAPI pictograms for SA-DLLME TLC-DIC: (**a**) for pharmaceutical samples, and (**b**) for biological samples.

The second assessment tool is GAPI, which was introduced by Płotka-Wasylka in 2018. In GAPI, 15 zones are distributed among five pentagrams in a three-colour pictogram. Each segment represents a phase in the analytical process, from sample collection to waste disposal. The ComplexGAPI pictogram generated for the proposed methodology is shown in Figure 4a,b. The colour of each pentagram (e.g., red, yellow, and green) signifies the level of environmental impact of each step during the analysis. In this manner, the final GAPI pictogram offers a complete and rapid overview of the greenness of the analytical method. Although the majority of the pentagrams in Figure 4a,b are either yellow or green, this illustrates compliance with the GAC principles. Hence, it is possible to infer that the proposed analytical approach is sufficiently green and has no negative impact on environmental and human safety. Figure 4a,b represent the location of the red pentagrams, at 5, 7 and 1, 3, 5, 7, respectively. These red pentagrams correspond to (1) sample collection (offline), (3) transportation (required), (5) extraction (required), and (7) usage of non-green solvent. According to the GAPI pictograms (Figure 4a,b), the main advantage of the proposed method is that no high-end analytical instrument is required for the analysis of FAV; thus, the fifth pentagram related to instrumentation (F12-F15) is not applicable in the case of SA-DLLME-TLC-SDIC.

**Figure 5:**
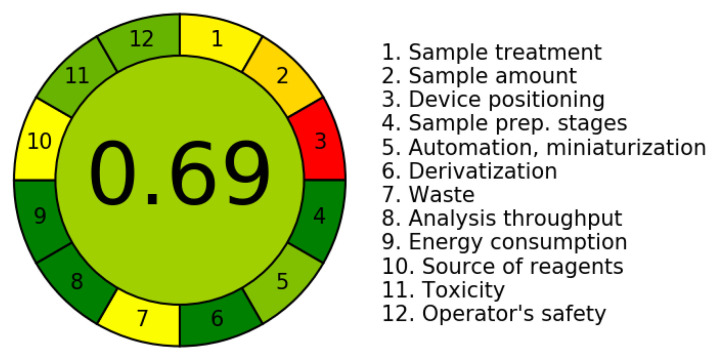
AGREE pictograms of the proposed method.

**Figure 6:**
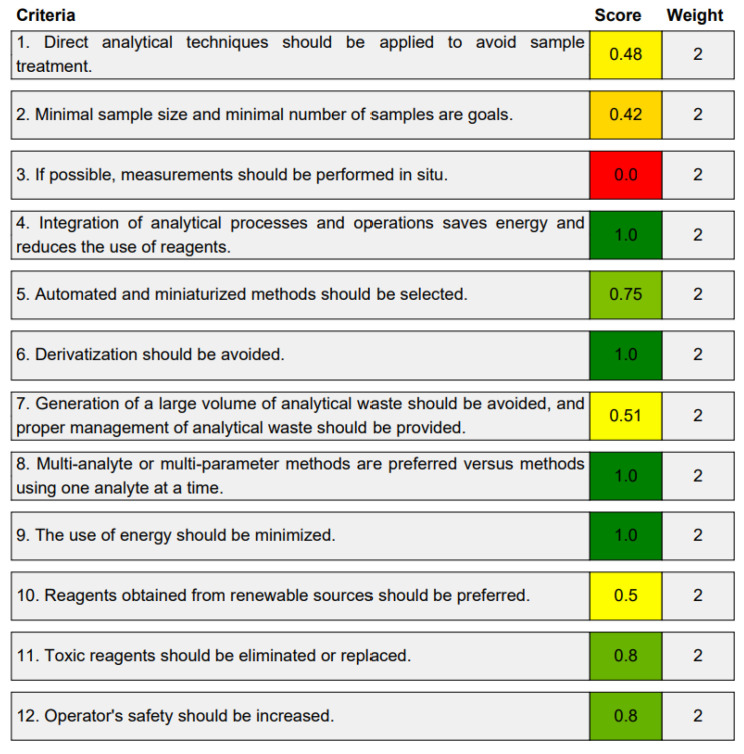
Greenness report sheet for the proposed method calculated by using 12 different greenness criteria with the AGREE software.

The third evaluation tool is the AGREE method, which was developed in 2020 by Pena-Pereira et al. This tool provides a colourful clock-shaped pictogram. Each portion of the perimeter represents one of the 12 guiding principles of GAC. In the centre of the AGREE pictogram, a score is displayed. A colour scheme that ranges from green to yellow to red is used to symbolise each parameter, which is given a score between 0 and 1. Greenness is higher if the number is significantly closer to 1. This tool is more focused on energy use and waste production rather than being concerned with the toxic impact of specific chemicals. Figure 5 and Figure 6 depict the overall AGREE score and the AGREE report sheets for the proposed analytical approach, respectively. It can be observed from Figure 5 and Figure 6 that the suggested approach obtained an overall AGREE score of 0.69, indicating that it is an excellent green method. Table 4 and Table 5 compare the suggested method to previously reported methods for identifying similar analytes based on greenness and various analytical parameters, respectively.

### 2.6. Application to Real Samples

Under optimised and validated conditions, the proposed method was successfully applied to quantify FAV in spiked biological matrices such as human urine and plasma samples, as well as in pharmaceutical formulations. Using the validated methods, the amount of FAV in relation to the indicated contents (400 and 800 mg/tablet) was also determined. In addition, Table 6 and Table 7 display the outcomes of applications of the proposed method for quantitative determination of FAV in biological matrices and pharmaceutical samples, respectively. Moreover, comparisons were made between the results obtained by the proposed method and by the standard HPLC method for biological matrices and pharmaceutical samples. The results from the two approaches were compared using a *t*-test with a 95% level of confidence and were found to be very close in agreement. There were no significant differences between the three sets of results, as the experimental *t*-values (−1.2, −1.5, 1.10, and 0.44) were lower than the crucial *t*-value (2.131 and 2.919) for *p* = 0.05.

## 3. Experimental Section

### 3.1. Reagents and Materials

Every reagent and chemical used in the research was of analytical grade unless otherwise specified. Surfactants such as Triton X-100, SDS (sodium dodecyl sulphate), and CTAB (cetyltrimethylammonium bromide) were procured from Sigma (USA). Chlorobenzene (CB) (purity > 99%) and dichloromethane (DCM) (purity > 99%) were obtained from Loba Chemie (Mumbai, India). Ethanol (EtOH) (purity > 99%), acetone (ACE) (purity > 99%), and methanol (MeOH) (purity > 99%) were acquired from Merck (Darmstadt, Germany). Chloroform (CF) (purity > 99%) and hydrochloric acid (HCl) (purity > 99%) were purchased from Thermo Fisher Scientific (Massachusetts, USA). Throughout the study, triple-distilled water was used. A standard of Favipiravir (FAV, purity > 99%) was purchased from the Indian Pharmacopoeia Commission (IPC, Ghaziabad, India) (lot no. IPRS/56/20). TLC silica gel 60 F_254_ pre-coated TLC aluminium plates (20×20 cm) were obtained from Merck (Darmstadt, Germany).

### 3.2. Sample Collection

Plasma samples was obtained by centrifuging the whole blood at 5000 rpm for 10 min. The whole blood was provided by the Rotary and Blood Bank Society Resource Centre, Chandigarh (India). Urine samples were obtained from three healthy volunteers aged between 28 and 40 years (two females and one male), who were also authors of this study. All of the biological samples were kept at ~4 °C and thawed at room temperature before analysis. Two distinct FAV tablets were procured from a local Chandigarh (India) market and labelled as having 800 and 400 mg of FAV per tablet, respectively.

### 3.3. Preparation of Standards and Samples

The stock solution of FAV was prepared at a concentration of 1 mg mL^−1^ in MeOH and stored at ~4 °C until needed. Working solutions of FAV at concentrations in the range of 5–100 µg/spot were prepared by appropriate dilution of stock solutions with ultrapure water. In order to imitate drug–protein binding under physiological conditions, biological samples such as urine and plasma were spiked with various amounts of FAV in the range of 5–100 µg/spot. These samples were then homogenised by vortex agitation for 5 min and incubated at 37 °C for 30 min [54].

### 3.4. Multivariate Analysis

Various experimental factors, including pH, volume of extraction and disperser solvents, sonication time, vortex agitation time, and vortex speed, which have significant impacts on DLLME extraction efficiency, were examined systematically. For this purpose, the following two-step strategy was utilised to optimise these factors using multivariate analysis: (i) Plackett–Burman design (PBD) to determine the significant parameters, and (ii) central composite design (CCD) to optimise the significant factors obtained by PBD. Multivariate analysis was carried out using the TIBCO STATISTICA software (Palo Alto CA, USA, Trial version).

### 3.5. SA-DLLME Procedure

#### 3.5.1. Pharmaceutical Formulations

Five tablets from each dose (i.e., 800 and 400 mg) were weighed and their average weight was calculated. These tablets were then crushed and converted into a fine powder. The equivalent to the average weight of the tablets was dissolved in 10 mL of MeOH for each dose (i.e., 800 and 400 mg) and then sonicated for 10 min. To achieve a concentration of 10 µg mL^−1^, the filtrate was appropriately diluted using ultrapure water. Under optimal conditions, 10 µg mL^−1^ of FAV was spiked into 5 mL of ultrapure water at a pH of 5 with the help of a 0.1 M HCl solution. Thereafter, a mixture of DCM (100 µL) and Triton X-100 (1.13 mmol L^−1^) was instantly injected into the aqueous sample. This stage resulted in the formation of a cloudy solution with tiny Triton X-100 droplets dispersed throughout the entire aqueous phase. In order to amplify the mass transfer of the analyte from the aqueous phase to the extraction solvent, the sample was sonicated for 130 s, followed by centrifugation at 5000 rpm for 3 min. After centrifugation, the sedimented phase was kept intact, while the supernatant was carefully discarded. The process of DLLME was completed within only 10 min. Subsequently, a TLC plate was spotted with 20 µL of the sedimented phase for further analysis.

#### 3.5.2. Biological Samples

Urine samples were initially centrifuged and filtered to remove any extra debris. Furthermore, 1 mL of blank biological samples (i.e., human urine and plasma) was fortified with 10 µg mL^−1^ of FAV and incubated for 30 min at 37 °C to stimulate drug–protein binding under physiological conditions. For the purpose of drug extraction, the biological samples were diluted to 5 mL with ultrapure water at pH 5. The samples were then subjected to the aforementioned DLLME procedure.

### 3.6. Thin-Layer Chromatography–Smartphone-Based Digital Image Colorimetry (TLC-SDIC) Procedure

**Figure 7:**
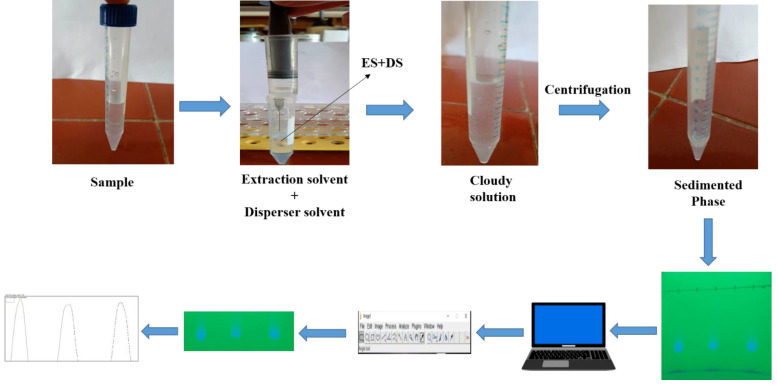
Systematic procedure of SA-DLLME-TLC-DIC for the determination of FAV.

With the aid of a micropipette, 20 µL (2 × 10 µL) of the sedimented phase obtained by DLLME was spotted on the marked start edge of the TLC plate (20 × 20 cm pre-coated silica gel 60 F254 aluminium-backed, purchased from Merck, Darmstadt, Germany) at a height of 1 cm. The TLC plate was put into a development chamber that had been pre-saturated with vapours of 10 mL of a mobile phase made up of CF:MeOH (8:2, *v*/*v*) after the spots were air-dried to remove any remaining solvent residues. Ascending mode was used to develop the plates up to 10 cm of solvent front migration away from the point of origin. Later, after 15–20 min of development, the TLC plate was taken out from the development chamber, allowed to air-dry, and then put in a UV cabinet at 254 nm for visualisation of spots. Under UV illumination, images of the developed TLC plates were captured with a smartphone camera. The FAV was visible as a blue spot against the light green background of the TLC plate. This image was transferred onto a laptop and saved in JPEG format. Furthermore, the image was split into R, B, and G channels (image > colour > split channel) using the freely available software ImageJ (National Institutes of Health, MD, USA). Since the green channel displayed the best sensitivity, it was chosen for quantitative analysis. The following steps were used to transform the spots of the G channel into peaks: (i) the “Rectangular selection tool” was used to select every spot at once; (ii) using the “plot lane tool”, peaks were plotted from all of the spots; (iii) a median filter with a resolution of 5–10 pixels was applied to remove noise from the peak; (iv) a line was drawn at the bottom of each peak using the “line tool”; and (v) by clicking inside the peak with the “magic wand tool”, the corresponding peak area was displayed. For statistical analysis, these peak areas were used (Figure 7). TLC densitograms of the standard, human urine, and plasma samples are depicted in Figure S3a–c, respectively.

### 3.7. Method Validation

The proposed method was validated for linearity, precision, recovery, and sensitivity as per the guidelines of the International Conference of Harmonisation (ICH) [55]. For method validation purposes, ultrapure water, human urine, and plasma samples were spiked in a range from 5 to 100 µg mL^−1^ (which is equal to 5–100 µg/spot). The linearity of the proposed method was evaluated by plotting calibration curves between peak areas (obtained by image analysis with ImageJ) and their corresponding concentrations on the *y*- and *x*-axes for aforementioned matrices. Linear regression analysis was used to determine the coefficient of determination (R^2^), slope, and intercept. The sensitivity of the proposed method was expressed as the limit of detection (LOD) and limit of quantification (LOQ). Furthermore, based on the assessment of the relative standard deviation (%RSD) at three different concentration levels of the calibration graph, the intraday and interday precisions (*n* = 5) were calculated. Additionally, the accuracy and relative recoveries at three different concentrations—including 20 µg/spot (low QC), 60 µg/spot (medium QC), and 100 µg/spot—were evaluated (high QC).

### 3.8. HPLC Analysis

A Shimadzu LC-2010 HT HPLC system with a UV detector was used for the chromatographic analysis. FAV was separated using a C18 column (5 µm film thickness, 250 mm length, 4.6 I.D.). A combination of 50 mM phosphate buffer (pH = 2.5) and acetonitrile at a ratio of 60:40 *v*/*v* was chosen as the mobile phase and pumped at a flow rate of 1 mL min^−1^ for the elution of the analyte of interest at a 30 ˚C column temperature. A wavelength of 323 nm was selected for the detection of FAV. After the DLLME procedure, the sedimented phase was completely evaporated and reconstituted in MeOH, 20 µL of which was subsequently injected into the HPLC system. The retention time of FAV was found to be 3.8 min. HPLC chromatograms of the standard, pharmaceutical, human urine, and human plasma samples are shown in Figure S4a–d, respectively.

## 4. Conclusions

The development of sustainable and green analytical protocols has attracted significant attention in the recent past. This has led to the replacement of hazardous organic solvents in order to reduce the risks to both humans and the environment. As a result, sample preparation is perfectly compatible with the principles of GAC. Herein, the present study proposes a simple, green, quick, high-sample-throughput, and cost-effective analytical approach for determining FAV in biological and pharmaceutical samples. The novel aspect of the developed method is the integration of SA-DLLME with TLC-SDIC, which provides a straightforward, instrument-less, and affordable analytical platform with the least consumption of electricity and minimal waste generation. With the help of SDIC, quantitative analysis can be carried out with a basic smartphone camera and open-source image processing software, without the need for any heavy, high-end instrumental techniques such as GC-MS or HPLC. The proposed procedures were evaluated using green metrics (i.e., the Analytical Eco-Scale, AGREE, and ComplexGAPI tools) and were found to be easier, sufficiently sensitive, and more environmentally friendly. The proposed method could be of immense use in forensic and clinical laboratories for medico-legal and pharmaceutical applications. Furthermore, this approach could serve as a stepping stone for the development of such analytical methods, which could be of immense use in resource-limited laboratories.

## Figures and Tables

**Table 1:** Analytical characteristics of the SA-DLLME TLC DIC method for FAV (*n* = 5).

Sample	LOD (µg/Spot)	LOQ (µg/Spot)	R^2^	Linearity (µg/Spot)		Precision (%RSD)					
					Calibration Curve	Intraday (µg/Spot)			Interday (µg/Spot)		
						20	60	100	20	60	100
Pharmaceutical formulation	1.2	3.96	0.9914	5–100	y = (471.92 ± 17.2) x + (32,100 ± 1022.70)	0.37	0.28	0.12	5.5	8.78	8.9
Urine	1.3	4.29	0.991	5–100	y = (610.88 ± 19.4) x + (40,665 ± 1151.152)	1.3	0.59	0.44	6.3	8.1	6.5
Plasma	1.5	4.95	0.994	5–100	y = (555.58 ± 12.00) x + (57,800 ± 710.39)	1.5	0.73	0.51	5.4	6.0	5.5

**Table 2:** Extraction efficiency parameters of the proposed method (*n* = 5).

Drug	Accuracy%						RR%	
	20 µg/Spot	60 µg/Spot	100 µg/Spot	EF	ER%	20 µg/Spot	60 µg/Spot	100 µg/Spot
Pharmaceutical formulation	102	102.3	96.6	53.9	107.8	90.1	96.2	98.2
Urine	97.1	103	102.3	46.0	92.0	92.5	89.5	97.5
Plasma	91.6	98.4	100.8	35.1	70.2	87.6	94.4	96

**Table 3:** Penalty points calculated by Analytical Eco-Scale score.

Reagents	Penalty Points
DCM	1 × 4 = 4
MeOH	1 × 6 = 6
CF	1 × 6 = 6
**Instrument**	
Energy used	0
Occupational hazard	0
Waste	0
**Total**	16
**Score**	84

**Table 4:** Comparison between the proposed method and previously reported methods for the determination of FAV.

Methods	Eco-Scale		GAPI	AGREE	Ref.
HPLC-UV	**Reagents**	**Penalty Points**	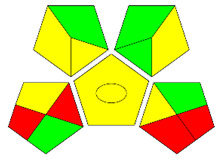	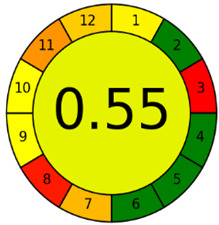	[10]
	MeOH	6			
	**Instrument**				
	Energy used	2			
	Waste	8			
	**Total**	16			
	**Score**	84			
HPLC-DAD	**Reagents**	**Penalty Points**	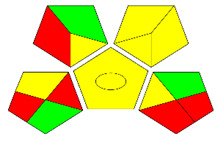	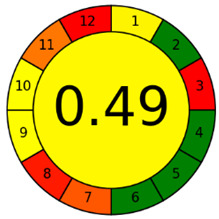	[49]
	ACN	8			
	**Instrument**				
	Energy used	2			
	Waste	8			
	**Total**	18			
	**Score**	82			
HPLC-UV	**Reagents**	**Penalty Points**	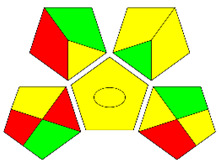	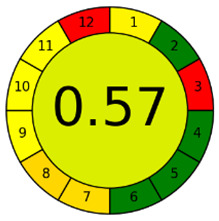	[50]
	ACN	8			
	**Instrument**				
	Energy used	2			
	Waste	8			
	**Total**	18			
	**Score**	82			
LC-MS/MS	**Reagents**	**Penalty Points**	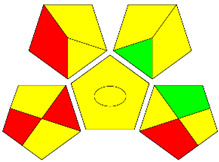	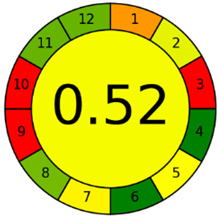	[4]
	MeOH Acetic acid	6 4			
	**Instrument**				
	Energy used	2			
	Waste	3			
	**Total**	15			
	**Score**	85			
LC-MS/MS	**Reagents**	**Penalty Points**	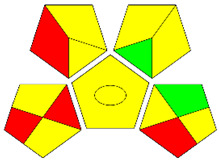	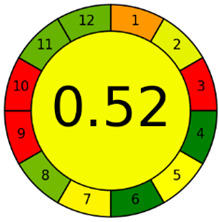	[20]
	ACN MeOH	8 6			
	**Instrument**				
	Energy used	2			
	Waste	3			
	**Total**	19			
	**Score**	81			
SA-DLLME-TLC-DIC	**Reagents**	**Penalty Points**	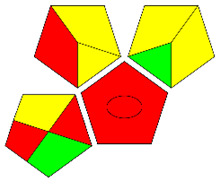	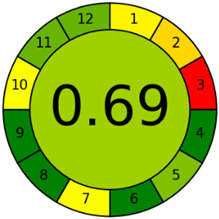	Present study
	MeOH DCM CF	6 4 6			
	**Instrument**				
	Energy used	0			
	Waste	0			
	**Total**	16			
	**Score**	84			

**Table 5:** Comparison of the proposed method with previously published methods for determining FAV in biological samples.

Sample Matrix	Sample Pre-Treatment and Extraction Method	Technique	Linearity Range	LOD	LOQ	Ref.
Spiked human plasma	Protein precipitation	Spectrofluorimetric method	40–280 ng mL^−1^	9.44 ng mL^−1^	28.60 ng mL^−1^	[14]
Human serum	SPE	LC-MS/MS	3291–20,790 μg L^−1^	-	3291 μg L^−1^	[51]
Human plasma	LLE	HPLC/UV	0.5–50 mg L^−1^	0.15 mg L^−1^	0.45 mg L^−1^	[52]
Human plasma	Protein precipitation	UPLC–MS/MS	0.25–16 μg mL^−1^	-	0.25 μg mL^−1^	[17]
Human urine	-	SW-AdSV	1.0–100.0 μg mL^–1^	0.26 μg mL^–1^	0.87 μg mL^–1^	[53]
Human plasma and urine	SA-DLLME	TLC-DIC	5–100 µg/spot	1.2–1.5 µg/spot	3.96–4.29 µg/spot	Present work

SW-AdSV: square-wave adsorptive stripping voltammetry.

**Table 6:** Determination of FAV in spiked biological samples (*n* = 5).

Samples	Concentration Prepared (µg/mL)	Concentration Found by HPLC Method (µg/mL)	Accuracy (%) by HPLC Method	Concentration Found by SA-DLLME TLC-DIC Method (µg/mL)	Accuracy (%) by SA-DLLME TLC-DIC Method
Urine	10	9.1	91	9.4	94
Plasma	10	9.6	96	9.8	98

**Table 7:** Determination of FAV in pharmaceutical formulations (*n* = 5).

Samples (Claimed FAV)	Concentration Prepared (µg/mL)	Concentration Found (µg/mL)	Accuracy with Respect to Claimed FAV (%)	Amount of FAV Found ^b^ (SA-DLLME TLC-DIC Method) ^b^	Amount of ASA/SA Found ^b^ (HPLC Method)
**800 mg**	10	9.3	99.4	795.2 ± 1.0	798.9 ± 2.4
**400 mg**	10	9.1	99.2	396.8 ± 1.3	398.9 ± 1.9

^b^ Data are expressed as the mean ± SD.

## Data Availability

Data available on reasonable request.

## Supplementary Materials

The following supporting information can be downloaded at: https://www.mdpi.com/article/10.3390/molecules28020529/s1, Figure S1: Pareto chart for standardized effects; Figure S2: Profiles for predicted values and desirability functions for peak responses of FAV. Vertical red line indicates current values after optimization; Figure S3: TLC densitogram of FAV obtained after SA-DLLME-TLC-SDIC (a) Standard, (b) Urine sample and (c) Plasma sample.; Figure S4: HPLC chromatogram of standard sample at 10 µg mL^−1^ (a); and pharmaceutical sample (b) Urine sample (c); and Plasma sample (d); Table S1: Factors and their levels tested in PBD; Table S2: Factors and their levels tested in CCD; Table S3: Experimental design matrix of CCD.

## References

[1] Mikhail I.E., Elmansi H., Belal F., Ibrahim A.E. (2021). Green micellar solvent-free HPLC and spectrofluorimetric determination of favipiravir as one of COVID-19 antiviral regimens. Microchem. J..

[2] Helmy Y.A., Fawzy M., Elaswad A., Sobieh A., Kenney S.P., Shehata A.A. (2020). The COVID-19 pandemic: A comprehensive review of taxonomy, genetics, epidemiology, diagnosis, treatment, and control. J. Clin. Med..

[3] Noureldeen D.A.M., Boushra J.M., Lashien A.S., Hakiem AF A., Attia T.Z. (2022). Novel environment friendly TLC-densitometric method for the determination of anti-coronavirus drugs “Remdesivir and Favipiravir”: Green assessment with application to pharmaceutical formulations and human plasma. Microchem. J..

[4] Morsy M.I., Nouman E.G., Abdallah Y.M., Zainelabdeen M.A., Darwish M.M., Hassan A.Y., Gouda A.S., Rezk M.R., Abdel-Megied A.M., Marzouk H.M. (2021). A novel LC-MS/MS method for determination of the potential antiviral candidate favipiravir for the emergency treatment of SARS-CoV-2 virus in human plasma: Application to a bioequivalence study in Egyptian human volunteers. J. Pharm. Biomed. Anal..

[5] Marzouk H.M., Rezk M.R., Gouda A.S., Abdel-Megied A.M. (2022). A novel stability-indicating HPLC-DAD method for determination of favipiravir, a potential antiviral drug for COVID-19 treatment; application to degradation kinetic studies and in-vitro dissolution profiling. Microchem. J..

[6] Abdallah I.A., Hammad S.F., Bedair A., Mansour F.R. (2022). Menthol-assisted homogenous liquid-liquid microextraction for HPLC/UV determination of favipiravir as an antiviral for COVID-19 in human plasma. J. Chromatogr. B: Anal. Technol. Biomed. Life Sci..

[7] Cai Q., Yang M., Liu D., Chen J., Shu D., Xia J., Liao X., Gu Y., Cai Q., Yang Y. (2020). Experimental Treatment with Favipiravir for COVID-19: An Open-Label Control Study. Engineering.

[8] Doi Y., Hibino M., Hase R., Yamamoto M., Kasamatsu Y., Hirose M., Mutoh Y. (2020). A Prospective, Randomized, Open-Label Trial of Early versus Late Favipiravir Therapy in Hospitalized Patients with COVID-19. Antimicrob. Agents Chemother..

[9] Furuta Y., Takahashi K., Kuno-Maekawa M., Sangawa H., Uehara S., Kozaki K., Nomura N., Egawa H., Shiraki K. (2005). Mechanism of action of T-705 against influenza virus. Antimicrob. Agents Chemother..

[10] Nguyen T.H.T., Guedj J., Anglaret X., Laouénan C., Madelain V., Taburet A.M., Baize S., Sissoko D., Pastorino B., Rodallec A. (2017). Favipiravir pharmacokinetics in Ebola-Infected patients of the JIKI trial reveals concentrations lower than targeted. PLoS Negl. Trop. Dis..

[11] Tkach V.V., Kushnir M.V., de Oliveira S.C., Ivanushko J.G., Velyka A.V., Molodianu A.F., Yagodynets P.I., Kormosh Z.O., dos Reis L.V., Luganska O.V. (2021). Theoretical description for anti-COVID-19 drug Remdesivir electrochemical determination, assisted by squaraine Dye–Ag2O2 composite. Biointerface Res. Appl. Chem..

[12] Mohamed M.A., Eldin G.M.G., Ismail S.M., Zine N., Elaissari A., Jaffrezic Renault N., Errachid A. (2021). Innovative electrochemical sensor for the precise determination of the new antiviral COVID-19 treatment Favipiravir in the presence of coadministered drugs. J. Electroanal. Chem..

[13] Allahverdiyeva S., Yunusoglu O., Yardım Y., Sentürk Z. (2021). First electrochemical evaluation of favipiravir used as an antiviral option in the treatment of COVID-19: A study of its enhanced voltammetric determination in cationic surfactant media using a boron-doped diamond electrode. Anal. Chim. Acta.

[14] Megahed S.M., Habib A.A., Hammad S.F., Kamal A.H. (2021). Experimental design approach for development of spectrofluorimetric method for determination of favipiravir; a potential therapeutic agent against COVID-19 virus: Application to spiked human plasma. Spectrochim. Acta Part A Mol. Biomol. Spectrosc..

[15] Raasi K.M. (2021). Analytical method development and validation of Remdesivir in bulk and pharmaceutical dosage forms using reverse-phase-high performance liquid chromatography. BR Nahata Smriti Sansthan Int. J. Phram. Sci. Clin. Res..

[16] Nguyen R., Goodell J.C., Shankarappa P.S., Zimmerman S., Yin T., Peer C.J., Figg W.D. (2021). Development and validation of a simple, selective, and sensitive LC-MS/ MS assay for the quantification of remdesivir in human plasma. J. Chromatogr. B.

[17] Rezk M.R., Badr K.A., Abdel-Naby N.S., Ayyad M.M. (2021). A novel, rapid and simple UPLC–MS/MS method for quantification of favipiravir in human plasma: Application to a bioequivalence study. Biomed. Chromatogr..

[18] Eryavuz Onmaz D., Abusoglu S., Onmaz M., Yerlikaya F.H., Unlu A. (2021). Development and validation of a sensitive, fast and simple LC-MS/MS method for the quantitation of favipiravir in human serum. J. Chromatogr. B.

[19] Xiao D., John Ling K.H., Tarnowski T., Humeniuk R., German P., Mathias A., Chu J., Chen Y.-S., van Ingen E. (2021). Validation of LC-MS/MS methods for determination of remdesivir and its metabolites GS-441524 and GS-704277 in acidified human plasma and their application in COVID-19 related clinical studies. Anal. Biochem..

[20] Avataneo V., de Nicolo A., Cusato J., Antonucci M., Manca A., Palermiti A., Waitt C., Walimbwa S., Lamorde M., di Perri G. (2020). Development and validation of a UHPLC-MS/MS method for quantification of the prodrug remdesivir and its metabolite GS-441524: A tool for clinical pharmacokinetics of SARS-CoV-2/COVID-19 and Ebola virus disease. J. Antimicrob. Chemother..

[21] Jain B., Jain R., Kabir A., Sharma S. (2022). Rapid Determination of Non-Steroidal Anti-Inflammatory Drugs in Urine Samples after In-Matrix Derivatization and Fabric Phase Sorptive Extraction-Gas Chromatography-Mass Spectrometry Analysis. Molecules.

[22] Sajid M., Płotka-Wasylka J. (2022). Green analytical chemistry metrics: A review. Talanta.

[23] Jain R., Singh R. (2016). Applications of dispersive liquid-liquid micro-extraction in forensic toxicology. TrAC-Trends Anal. Chem..

[24] Jain R., Singh R. (2021). Microextraction Techniques in Analytical Toxicology.

[25] Jain R., Singh R. (2016). Microextraction techniques for analysis of cannabinoids. TrAC-Trends in Analytical Chemistry.

[26] Jha R.R., Thakur R.S., Jain R. (2022). Dispersive Liquid-Liquid Microextraction and Its Variants. Microextraction Techniques in Analytical Toxicology.

[27] Mudiam MK R., Jain R., Singh A., Khan H.A., Parmar D. (2014). Development of ultrasound-assisted dispersive liquid-liquid microextraction-large volume injection-gas chromatography-tandem mass spectrometry method for determination of pyrethroid metabolites in brain of cypermethrin-treated rats. Forensic Toxicol..

[28] Jain R., Singh M., Kumari A., Tripathi R.M. (2021). A rapid and cost- effective method based on dispersive liquid- liquid microextraction coupled to injection port silylation-gas chromatography- mass spectrometry for determination of morphine in illicit opium. Anal. Sci. Adv..

[29] Olędzka I., Kowalski P., Plenis A., Bączek T. (2017). Evaluation of various approaches to the isolation of steroid hormones from urine samples prior to FASS-MEKC analysis. Electrophoresis.

[30] Primel E.G., Caldas S.S., Marube L.C., Escarrone A.L.V. (2017). An overview of advances in dispersive liquid–liquid microextraction for the extraction of pesticides and emerging contaminants from environmental samples. Trends in Environmental Analytical Chemistry.

[31] Jain R., Jha R.R., Kumari A., Khatri I. (2021). Dispersive liquid liquid microextraction combined with digital image colorimetry for paracetamol analysis. Microchem. J..

[32] Jain B., Jain R., Jha R.R., Bajaj A., Sharma S. (2022). A green analytical approach based on smartphone digital image colorimetry for aspirin and salicylic acid analysis. Green Anal. Chem..

[33] Jain R., Singh R., Sudhaker S., Barik A.K. (2017). Coupling Microextraction with Thin Layer Chromatography-Image Processing Analysis: A New Analytical Platform for Drug Analysis. Toxicol. Forensic Med..

[34] Jain R., Kumari A., Khatri I. (2021). Simple and rapid analysis of acetaminophen in human autopsy samples by vortex-assisted dispersive liquid–liquid microextraction-thin layer chromatography-image analysis. Sep. Sci. Plus.

[35] Jain R., Tripathi R.M., Negi A., Singh S.P. (2020). A simple, cost-effective and rapid method for simultaneous determination of Strychnosnux-vomica alkaloids in blood and Ayurvedic medicines based on ultrasound-assisted dispersive liquid-liquid microextraction-thin-layer chromatography-image analysis. J. Chromatogr. Sci..

[36] Meisen I., Wisholzer S., Soltwisch J., Dreisewerd K., Mormann M., Müthing J., Karch H., Friedrich A.W. (2010). Normal silica gel and reversed phase thin-layer chromatography coupled with UV spectroscopy and IR-MALDI-o-TOF-MS for the detection of tetracycline antibiotics. Anal. Bioanal. Chem..

[37] Parys W., Pyka-Pająk A. (2022). TLC–Densitometry for Determination of Omeprazole in Simple and Combined Pharmaceutical Preparations. Pharmaceuticals.

[38] Domínguez C., Jover E., Garde F., Bayona J.M., Erra P. (2003). Characterization of Supercritical Fluid Extracts from Raw Wool by TLC-FID and GC-MS. J. Am. Oil Chem. Soc..

[39] Nakamura K., Suzuki Y., Goto-Inoue N., Yoshida-Noro C., Suzuki A. (2006). Structural characterization of neutral glycosphingolipids by thin-layer chromatography coupled to matrix-assisted laser desorption/ionization quadrupole ion trap time-of-flight MS/MS. Anal. Chem..

[40] Olech M., Komsta L., Nowak R., Ciesla L., Hajnos M.W. (2012). Investigation of antiradical activity of plant material by thin-layer chromatography with image processing. Food Chem..

[41] Skowron M., Zakrzewski R., Ciesielski W. (2016). Application of thin-layer chromatography image analysis technique in quantitative determination of Sphingomyelin. J. Anal. Chem..

[42] Moffat A.C., Osselton M.D., Widdop B., Watts J. (2011). Clarke’s Analysis of Drugs and Poisons.

[43] Fried B. (1982). Thin-layer chromatography: Techniques and applications. Pascal Fr. Bibliogr. Databases.

[44] Ghaedi M., Roosta M., Khodadoust S., Daneshfar A. (2015). Application of optimized vortex-assisted surfactant-enhanced DLLME for preconcentration of thymol and carvacrol, and their determination by HPLC-UV: Response surface methodology. J. Chromatogr. Sci..

[45] Moradi M., Yamini Y., Esrafili A., Seidi S. (2010). Application of surfactant assisted dispersive liquid-liquid microextraction for sample preparation of chlorophenols in water samples. Talanta.

[46] Hemdan A., Magdy R., Farouk M., Fares N.v. (2022). Central composite design as an analytical optimization tool for the development of eco-friendly HPLC-PDA methods for two antihypertensive mixtures containing the angiotensin receptor blocker Valsartan: Greenness assessment by four evaluation tools. Microchem. J..

[47] Jain B., Jain R., Jha R.R., Ghosh A., Basu D., Abourehab MA S., Bajaj A., Chauhan V., Kaur S., Sharma S. (2023). Cellulose paper sorptive extraction (CPSE): A simple and affordable microextraction method for analysis of basic drugs in blood as a proof of concept. J. Chromatogr. B.

[48] Jain R., Jain B., Chauhan V., Deswal B., Kaur S., Sharma S., Abourehab M.A.S. (2023). Simple determination of dichlorvos in cases of fatal intoxication by gas Chromatography-Mass spectrometry. J. Chromatogr. B.

[49] Feng G., Ding W., Deng Y., Zhao R., Gong Y., Duan C., Sun J. (2015). A Kind of Favipiravir Has the HPLC Assay Method of Related Substance.

[50] Bulduk I. (2021). HPLC-UV method for quantification of favipiravir in pharmaceutical formulations. Acta Chromatogr..

[51] Habler K., Brügel M., Teupser D., Liebchen U., Scharf C., Schönermarck U., Vogeser M., Paal M. (2021). Simultaneous quantification of seven repurposed COVID-19 drugs remdesivir (plus metabolite GS-441524), chloroquine, hydroxychloroquine, lopinavir, ritonavir, favipiravir and azithromycin by a two-dimensional isotope dilution LC–MS/MS method in human serum. J. Pharm. Biomed. Anal..

[52] Timofeeva I., Kanashina D., Kirsanov D., Bulatov A. (2018). A heating-assisted liquid-liquid microextraction approach using menthol: Separation of benzoic acid in juice samples followed by HPLC-UV determination. J. Mol. Liq..

[53] Akça Z., Özok H İ., Yardim Y., Şentürk Z. (2022). Electroanalytical investigation and voltammetric quantification of antiviral drug favipiravir in the pharmaceutical formulation and urine sample using a glassy carbon electrode in anionic surfactant media. Turk. J. Chem..

[54] Urso A.D., Rudge J., Patsalos P.N., De Grazia U. (2019). Volumetric Absorptive Microsampling: A New Sampling Tool for Therapeutic Drug Monitoring of Antiepileptic Drugs. Ther. Drug Monit..

[55] Teasdale A., Elder D., Nims R.W. (2005). ICH Topic Q2(R), Validation of Analytical Procedure: Methodology. ICH Harmonized Tripartite Guidelines.

