# Supplementary material for: A Non-Instrumental Green Analytical Method Based on Surfactant-Assisted Dispersive Liquid–Liquid Microextraction–Thin-Layer Chromatography–Smartphone-Based Digital Image Colorimetry(SA-DLLME-TLC-SDIC) for Determining Favipiravir in Biological Samples
Source: Molecules. 2023 Jan 5;28(2):529. doi: 10.3390/molecules28020529 (PMC9860899; doi:10.3390/molecules28020529)
Supplement: Supplementary file 1 [file molecules-28-00529-s001.zip › molecules-2126818-supplementary.pdf]

A Non-Instrumental Green Analytical Method Based on Surfactant-Assisted Dispersive Liquid–Liquid Microextraction–Thin-Layer Chromatography–Smartphone-Based Digital Image Colorimetry(SA-DLLME-TLC-SDIC) for Determining Favipiravir in Biological Samples

Bharti Jain [1,2,†], Rajeev Jain [1,*,†], Prashant Kumar Jaiswal [3], Torki Zughaibi [4,5], Tanvi Sharma [2], Abuzar Kabir [6,7], Ritu Singh [3] and Shweta Sharma [2,*]

[1] Central Forensic Science Laboratory, Dakshin Marg, Sector—36A, Chandigarh 160036, India
[2] Institute of Forensic Science & Criminology, Panjab University, Chandigarh 160014, India
[3] School of Earth Sciences, Department of Environmental Sciences, Central University of Rajasthan, NH-8, Bandar Sindri, Ajmer 305817, India
[4] Department of Medical Laboratory Sciences, Faculty of Applied Medical Sciences, King Abdulaziz University, Jeddah 21589, Saudi Arabia
[5] King Fahd Medical Research Center, King Abdulaziz University, Jeddah 21589, Saudi Arabia
[6] International Forensic Research Institute, Department of Chemistry and Biochemistry, Florida International University, Miami, FL 33199, USA
[7] Department of Pharmacy, Faculty of Allied Health Science, Daffodil International University, Dhaka 1207, Bangladesh
* Correspondence: rajeev.jain-as@gov.in or rajeevjaincfsl@gmail.com (R.J.); 25shweta@pu.ac.in (S.S.)
† These authors contributed equally to this work.

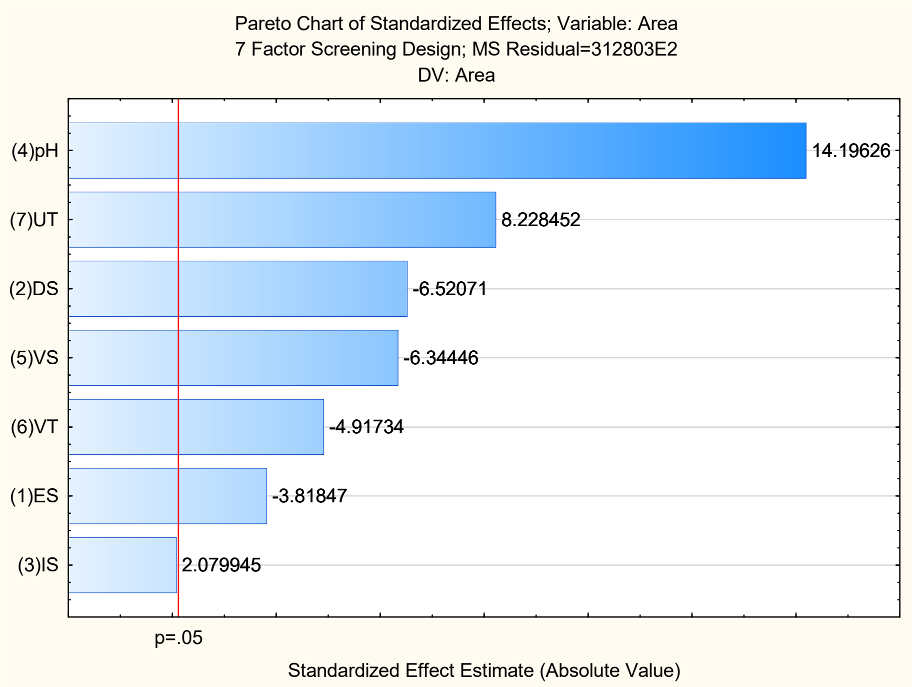

Figure S1: Pareto chart for standardized effects.

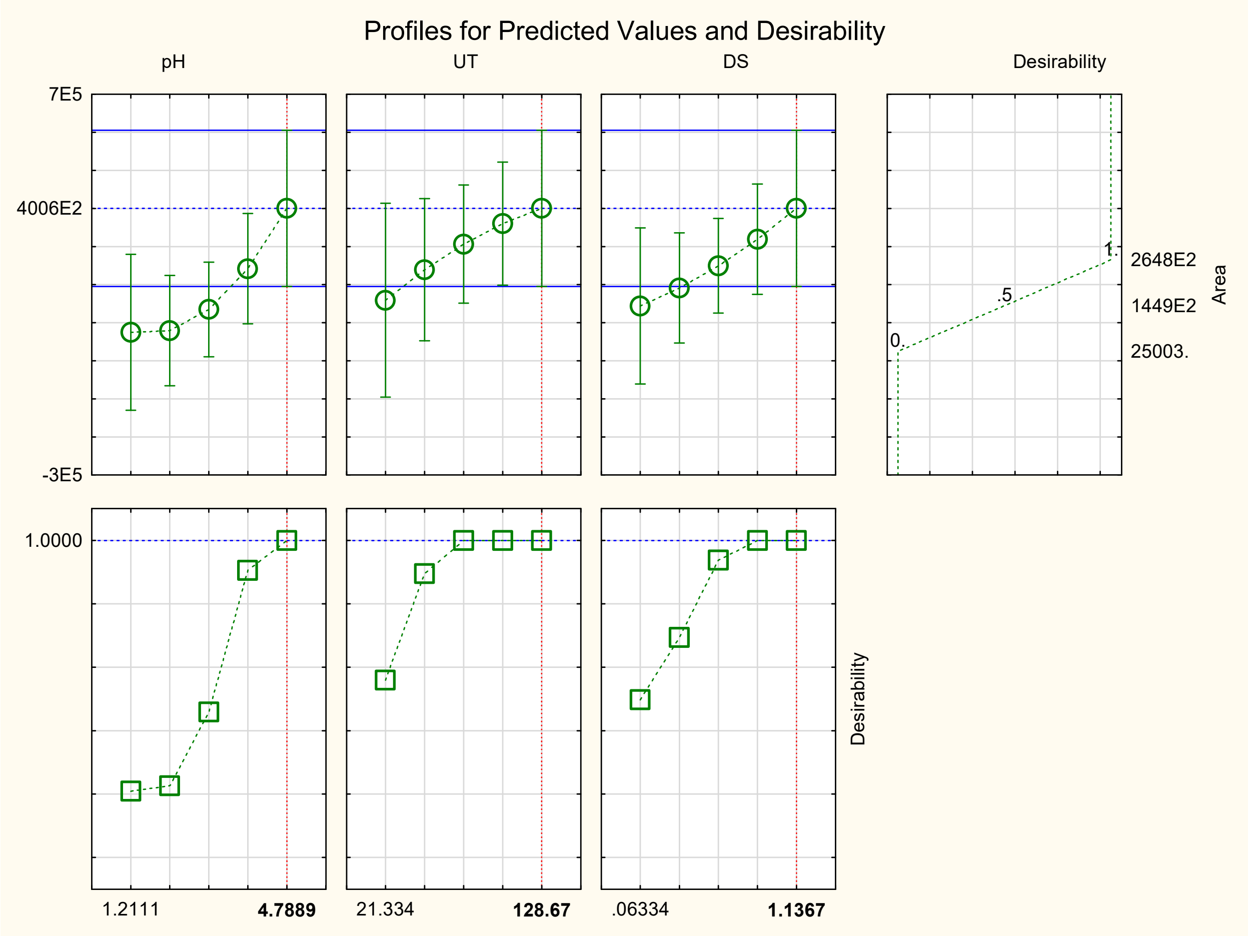

Figure S2: Profiles for predicted values and desirability functions for peak responses of FAV. Vertical red line indicates current values after optimization.

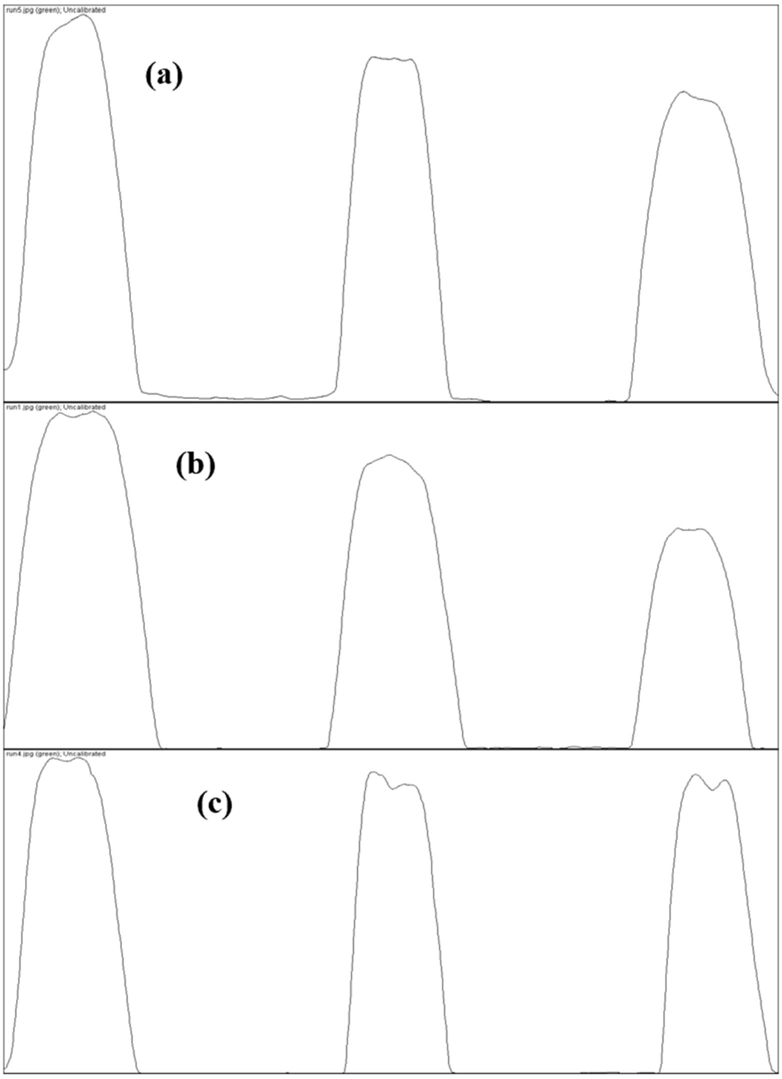

Figure S3: TLC densitogram of FAV obtained after SA-DLLME-TLC-SDIC (a) Standard, (b) Urine sample and (c) Plasma sample.

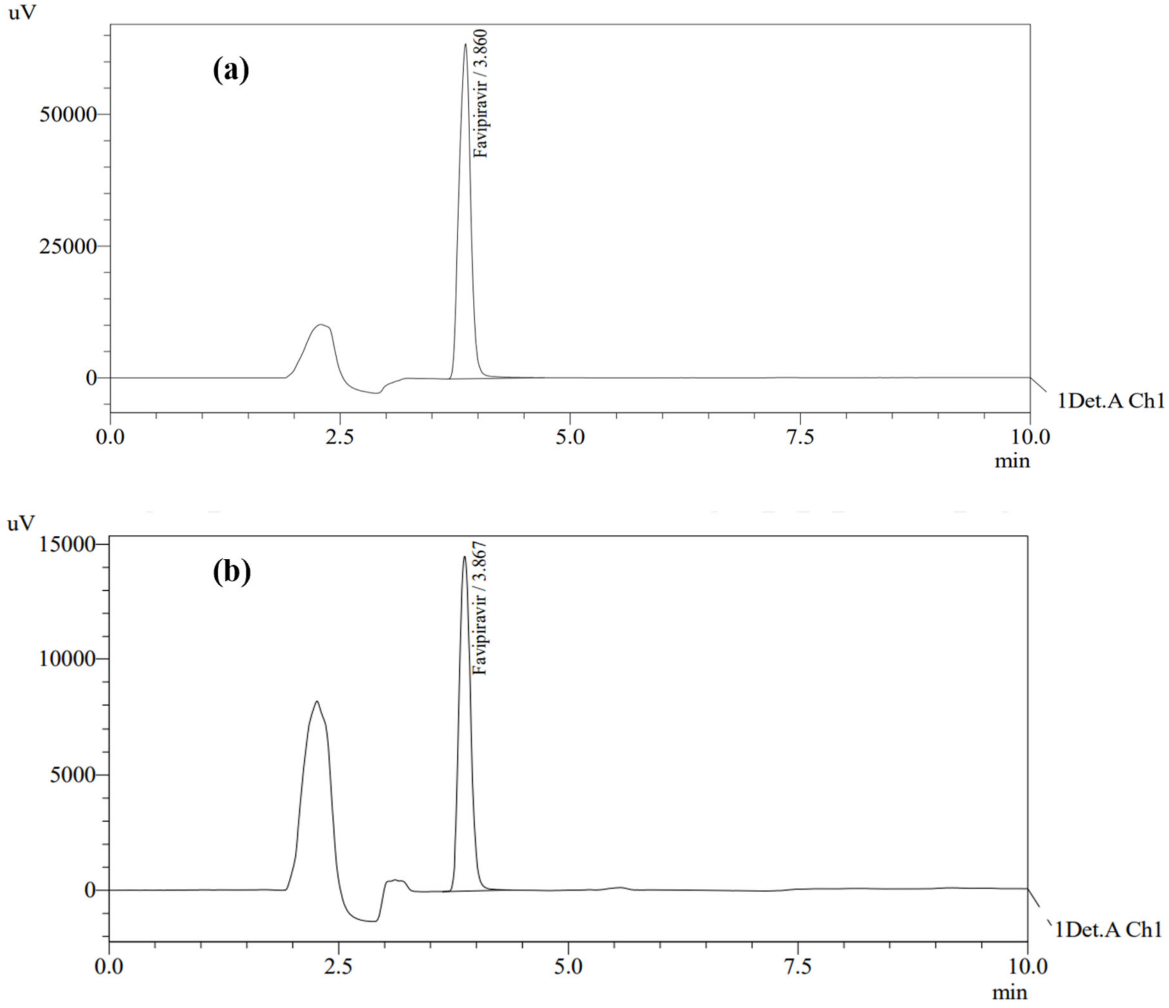

uV
(a)
Favipiravir / 3.860
50000
25000
0
0.0
2.5
5.0
7.5
10.0
min
1Det.A Ch1
uV
15000
(b)
Favipiravir / 3.867
10000
5000
0
0.0
2.5
5.0
7.5
10.0
min
1Det.A Ch1

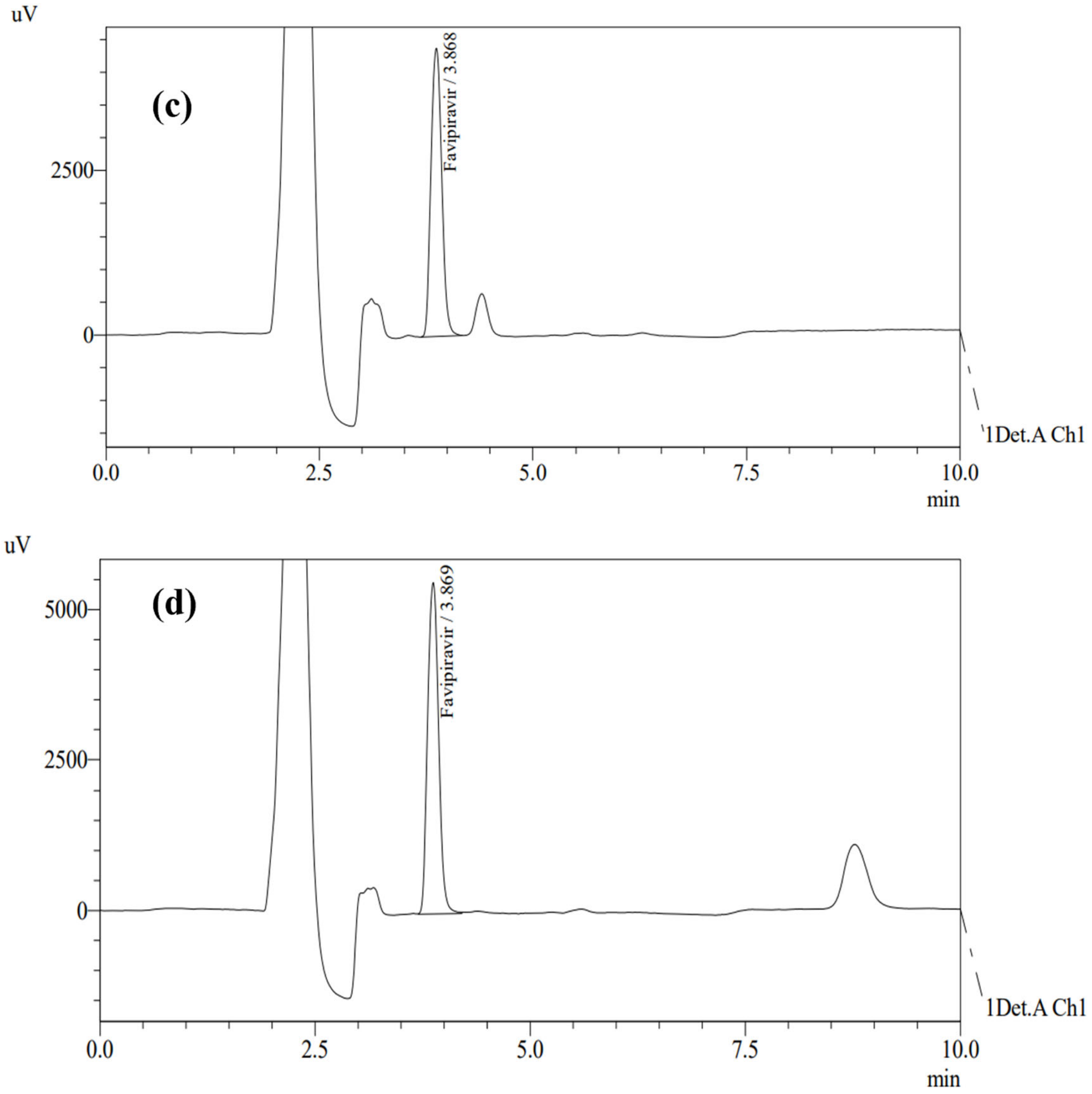

Figure S4: HPLC chromatogram of standard sample at 10 µg mL^{-1}(a); and pharmaceutical sample (b); HPLC chromatogram of Urine sample (c); and Plasma sample (d).

Table S1: Factors and their levels tested in PBD.

Factors	Levels	
	Low (-1)	High (+1)
Volume of extraction solvent (µL)	100	300
Volume of disperser solvent (m mol L^{-1})	0.20	0.3
Ionic strength (% NaCl)	5	10
pH	3	8
Vortex timing (sec.)	30	120
Vortex speed (rpm)	1000	3000
Ultrasonic time (sec.)	30	120

Table S2: Factors and their levels tested in CCD.

Factors	Levels			Star points	
	Low (-1)	Central (0)	High (+1)	-α	+α
pH	2	3	4	1.2	4.7
Ultrasonic time (sec.)	60	75	120	21	128
Volume of disperser solvent (m mol L^{-1})	0.30	0.60	0.90	0.06	1.13

Table S3: Experimental design matrix of CCD.

Standard run	Factors		
	pH	Ultrasonic time	Volume of disperser solvent
1	-1	-1	-1
2	-1	-1	+1
3	-1	+1	-1
4	-1	+1	+1
5	+1	-1	-1
6	+1	-1	+1
7	+1	+1	-1
8	+1	+1	+1
9 (C)	0	0	0
10 (C)	0	0	0
11	-α	0	0
12	+α	0	0
13	0	-α	0
14	0	+α	0
15	0	0	-α
16	0	0	+α
17 (C)	0	0	0
18 (C)	0	0	0
